# Cases of Tic Douloureux

**Published:** 1849-04

**Authors:** S. P. Hullihen


From the American Journal of Dental Science.

### CASES OF TIC DOULOUREUX

### SUCCESSFULLY TREATED WITH LUNAR CAUSTIC, BY APPLYING IT IN THE ANTRUM MAXILLARE, &C.; DESCRIBED IN A PAPER READ BEFORE THE OHIO CO. MEDICAL SOCIETY OF VIRGINIA.

BY DR. S. P. HULLIHEN.

MR. PRESIDENT:—I propose to offer a few observations on the effects of lunar caustic, when applied over the situations of painful nerves, as noticed during the treatment of several cases of tic douloureux.

Having observed that certain diseased conditions of the antrum maxillare induced tic douloureux, and that in all such cases the painful paroxysms could be greatly soothed or aggravated by the kind of injections thrown into the antrum—that of all the injections so employed, none had so distinct, so powerful, and so extensive an effect as lunar caustic; and knowing, too, that lunar caustic had been sometimes applied over the eyelids and brows, with the happiest effect in allaying pain and undue irritability of the eyes, I determined to try it in the treatment of true tic douloureux. I say *true* tic douloureux, a rare disease, emanating from some local cause either about the head or neck, in contradistinction to a *spurious* tic douloureux of the face, a complaint which is so frequently met with, and comparatively so easily cured; but a complaint always induced by debility, malaria, or other causes of a character purely constitutional.

But it is not my present intention to treat of the causes, nor of the pathological differences between the true and the spurious tic douloureux, nor of the still greater difference in the symptoms of the two diseases; but simply to give in detail the treatment of several cases of true tic douloureux, wherein the effects of lunar caustic will be described, and occasionally contrasted with the effects of other remedies employed in the same cases.

In the summer of 1844, Mr. J——, of Marshall county, Va., came to Wheeling, to obtain relief from an unusually severe attack of tic douloureux. He was about forty years of age; his occupation was that of a farmer, and his health good. The length of time he had been affected with the disease I neglected to note down. The nerves involved were the first and slightly the second branch of the fifth pair. The paroxysms came on from touching the affected side—often while talking or eating—and very frequently without being provoked by either of the causes just named. The attacks were electric in their character, accompanied by sensations of a *tic*—a symptom never present, I believe, in ordinary neuralgia. The paroxysms were of about one minute's duration, occurring many times every day and night; and they were gradually becoming more exquisitely painful.

Treatment.—I extracted the first molar tooth, it being decayed, and perforated the antrum by way of an alveolar cell which led directly to this cavity. The antrum was free from disease. Upon touching the outer wall of the cavity with the end of a probe, particularly if the end of the probe were dragged over the surface, paroxysms of pain were instantly induced. After the bleeding subsided, I washed out the antrum with a syringe, first with warm water and then with cold, and so, alternately, until I was convinced that warm water, in this particular case, had much agency in bringing on a paroxysm, and cold water as great an agency in allaying it. After the blood was thoroughly cleansed from the antrum, I threw into it, with a glass syringe, a solution of lunar caustic, (twenty-five grains to the ounce of water,) and there retained it for a few minutes, by plugging up the hole made in the jaw. The caustic had but little effect in any way. The next morning I increased the strength of the solution to fifty grains of caustic to the ounce of water. After taking up in the syringe about as much of the solution as the antrum would hold—the patient being directed to hold his head in a horizontal position, with the affected side down—it was injected into the antrum, and the opening stopped as before. In a few minutes the patient complained, first of a slight pain on the top of his head—then all over the side of his head—then over the eye, and finally in the antrum. The plug was now removed, and the solution suffered to escape into his mouth, his mouth being effectually protected by holding in it a solution of common salt.

By this time the effects of the treatment were visible. The veins of the affected side, particularly along the temple, were distended and elevated to a remarkable extent; tears streamed from the eye; the flow of saliva was unusual; indeed, every secreting vessel of that side of the head appeared to be excited in the highest possible degree; yet the patient complained of but little pain, and that pain of a dull, benumbing description. The scalp, and indeed the whole side of the head upon which the first and second branches of the fifth pair of nerves are distributed, was sore to the touch; but the patient was entirely free from every symptom of tic douloureux. He was now allowed to return home, and directed to wash out the antrum with cold water once a day—to use the caustic injection once a week—

and to return again in three or four weeks; which he did, and reported that he never had the slightest return of the disease after he left Wheeling. I saw him about five months since, and he still remained well.

It was not until October 20, 1846, that I had another opportunity of testing the good effects of lunar caustic in a case of tic douloureux. At that time Mr. Johnston, of this city brought to my office an old gentleman by the name of C——, who stated that he had been very sorely afflicted with tic douloureux for upwards of seven years; during which time he had been almost constantly under medical treatment, without any perceptible relief; that still his sufferings were so intolerable, he could not refrain from taking the medicine of every physician who would give him the least encouragement; and that he had now come to Wheeling for the purpose of getting the affected nerves divided, or to undergo any course of treatment that would promise relief.

The patient was about sixty years old—in excellent health—of slender form—intelligent and very communicative. He stated that in early life he had been a soldier; after which a surveyor; then a merchant; then a farmer; and then a victim to tic douloureux. He stated that he had always enjoyed good health; that the disease came upon him without any known cause; that the first symptom was an unpleasant twitching of the eyelid; then of the muscles of his face—then of a dull pain over his eye; and then, suddenly, the disease in full force. The paroxysms were electric in their character, usually preceded by and ending in numerous sensations of a *tic.* They were from half a minute to a minute and a half in duration, and were repeated from fifty to a hundred times every twenty-four hours. The nerves most implicated were the first and second branches of the fifth pair; the second was more affected than the first, and I was not certain but that the third branch was involved also.

Treatment.—I perforated the floor of the antrum, and after examining into the condition of the cavity, and finding it free from disease, I washed it out well, first with cold and then with warm water. Both appeared to provoke paroxysms of pain, yet the warm water caused more severe paroxysms than the cold. I then injected the antrum with a solution of lunar caustic, of fifty grains to the ounce of water, after the same manner described in the first case. However, before I could get the hole well plugged in the jaw, a most fearful paroxysm of the disease ensued, which was soon succeeded by another, and another, until the poor old man was nearly exhausted. In about ten minutes, this fearful condition of things subsided. I then increased the strength of the injection to sixty grains to the ounce, and threw a portion of it into the antrum, and there retained it by a plug. Three or four slight paroxysms of pain took place within the first five minutes; then the patient began to complain of a sensation of heat in the antrum; then of pain on the top of his head, and along the temple and over the eye, particularly over the eye, and, finally, in the cheek. The veins along the temple were distended, as in the former case; the secretion of tears small; the conjunctiva of a bright pink color, and the flow of saliva not much increased. The plug was now withdrawn, and the solution allowed to run out, its effects being neutralized in the mouth by the use of salt and water. The patient now complained of a dull, heavy, and distressing pain all over the affected side, and occasionally a slight sharp pain, something like tic douloureux; but this was all that was felt of the disease. The next day, the pain on the side of his head had subsided; there was much soreness of the scalp and temple; some congestion of the conjunctiva; a slight swelling of the cheek; and every now and then a very mild sensation of his old complaint. The next day, the soreness of that side of his head was better; he had slept well, and eat without *producing any return of his disease*, except an occasional darting pain through the cheek. On the fourth day, the patient was still getting better; matter had begun to be discharged from the antrum. On the fifth day, still improving; has every now and then a sharp pain in his cheek, just in front of his ear, but of a comparatively mild character. I now injected his antrum with a solution of sixty grains to the ounce of water. Again he had pain all over the side of his head; again the conjunctiva became flushed, and the scalp sore to the touch; but every symptom of disease had vanished. He left town the following day, with a swelled cheek, a sore scalp, but with a light heart. He was directed to wash out the antrum every day, with water and a little soap, to use the caustic solution once a week, and to let me hear from him in two or three weeks. At the end of two months, his son called to say that his father still remained well; that he had occasional twitchings of the muscles of the affected side of his head, but no pain; and that, should the disease return, he would come to Wheeling immediately. He has never come, from which I conclude he still remains well.

The treatment of the next case of tic douloureux I have to lay before you, is of more interest, by far, than either of the preceding ones. The patient was one of our own citizens, and for the last fifteen years, had been a perfect martyr to the disease. You have all seen him, and many of you have doubtless prescribed for him. I mean old Mr. P——. He is now upwards of seventy-five years old, and never had any severe sickness until 1833. He was then taken with the cholera in a very severe manner, from which he recovered but slowly. After he had regained his health and strength to a very considerable extent, he experienced for some time a singular, numb-like sensation in his face, which was followed by true tic douloureux. He immediately applied to his physician, was treated very energetically and perseveringly, without benefit. He then placed himself under the care of another physician, without any better result, and then under the care of another, and then another, until every physician who would give the old man the least hope, had an opportunity of trying his skill. In 1838, I had the old gentleman under own my care, without being able to afford him any relief, save what was obtained by the division of the mental nerve. Thus did the patient suffer and doctor for fifteen years, but still he had tic douloureux, and that, too, in its worst form.

On the 26th day of February, 1847, he called at my office, and begged to have something done that would give him a little relief. He stated that the paroxysms of pain were greatly more severe and frequent than common; that he could not speak, eat, nor even touch his face, without bringing on the pain; that he was also suffering from asthma, and a cough; that the cough troubled him most at night, and that every time he coughed, a paroxysm of pain ensued.

The nerves involved were the first, second, and third branches of the fifth pair. The first and second branches more than the third. The paroxysms were electric in their character, would continue from half a minute to two minutes, and occurred from twenty-five to one hundred and fifty times every twenty-four hours. The disease was always more severe in *cold* weather than in *warm*.

Treatment.—I perforated the floor of the antrum, and finding the cavity was free from disease, I injected into it a solution of lunar caustic of fifty grains to the ounce of water, and there confined it in the manner described in the former cases. In about a minute, a very severe paroxysm took place; soon after, another less severe; and then another still more mild. About this time, the patient began to complain of pain on the top of his head, then along the temple and over the eye, and then in the antrum. The plug was now removed from the jaw, and the solution suffered to escape.

*February 27th.*—The patient complains of a continuous pain all over the affected side of his head; some soreness; some sharp shooting pains, and every now and then a severe spell of his disease, coming on generally after a fit of coughing. I examined his throat, snipped off a portion of the uvula, it being too long, washed out the antrum with cold water, and directed him to return next day.

*28th.*—The pain and soreness of the side of the head is much abated; uvula some swelled, and very sore; the paroxysms of tic douloureux much less severe, and less frequent.

*29th.*—The patient about the same as yesterday.

*March 1st.*—No improvement. I now injected the antrum with a solution of lunar caustic of sixty grains to the ounce of water. In a few moments, the patient complained of pain on the top and side of his head, then of great heat in the antrum. Yet I continued to keep the solution stopped up in the cavity for fifteen or twenty minutes, and that, too, without materially increasing the pain in the temple and scalp.

2*d.*—Great soreness of the scalp, and along the temple; considerable swelling of the cheek; has no other symptoms of his complaint, except a ticking sensation, every now and then a twitching of the muscles, and occasionally a pain of an instant, darting from the front part of the ear over the masseter muscle, towards the corner of the mouth. Can eat and sleep well.

3*d.*—Soreness of the scalp and swelling of the cheeks still continue, as do also the *painless tic*, and the darting pain in front of the ear. The antrum was washed out with cold water, which occasioned much aching in the jaw.

4*th.*—Patient about the same as yesterday. Washed the antrum with warm water, without causing any unpleasant sensation.

6*th.*—Soreness of the scalp and swelling of the cheeks subsiding; matter discharging from the antrum; still feels the *tic* in the cheek, and still has the sharp pain over the belly of the masseter muscle. Injected the antrum with water.

9*th.*—Done nothing for the last three days, save injecting the antrum with warm water. Soreness and swelling of the cheek and scalp completely disappeared; still the patient feels the *tic* and the darting pain. But the *tic* and pain are not felt in the same region of the face. In this way the case remained, without any further improvement, although the same treatment with caustic, as before described, was repeated twice up to May 20th. On this day the patient had a slight return of his disease; although not so severe or frequent as formerly, yet he was amazingly alarmed. I now tried *creosote* in the antrum, of various degrees of strength, but without any effect. I then gave *morphia* a fair trial, by injecting a solution into the antrum, without producing any other effect than putting the old man very fast asleep upon two or three occasions. I also tried *belladonna*

in the same way, without producing any other effect than enlarging the pupil enormously, and keeping it so enlarged for nearly two days. I also tried several other articles that had some reputation in relieving tic douloureux, but all to no purpose, except the *spts. turpentine* and *ammonia.* This appeared to have a good effect, but the effect would soon pass off, and the disease return in full force.

*June 4th.*—The severity of the disease is increasing. I now blew into the antrum, from a glass tube, twenty-five grains of pulverized lunar caustic. Again the scalp became sore, and the face swelled; but all symptoms of the disease at once subsided, save the *tic* and the darting pain over the masseter muscle.

*7th.*—The patient still remains free from every symptom of tic douloureux, except the darting pain in front of the ear. I now made an external application of lunar caustic, about two inches broad, extending from the ear to the corner of the mouth.

*9th.*—Has not had the slightest pain of any description in his cheeks since the application of the caustic. Still the *tic* continued, and I was unable to remove this sensation by any course of treatment I could adopt. In this way the patient remained for five months. Then upon a warm day in the month of November, after much exercise, the patient seated himself in a hall, and fell asleep; upon waking, he had a stiff neck and shoulder; in short, he had taken cold. On the following day, he had two or three pretty severe paroxysms of tic douloureux. He now called upon me again. I again blew into the antrum, from a glass tube, thirty grains of pulverized lunar caustic, and plugged up the hole in the jaw. I also applied the caustic over the cheek as before. The cheek now became much more swollen than upon former occasions. The soreness of the scalp was also far greater, as was the pain upon the side of the face. The next day I washed out the antrum with salt and water. In a few days the swelling of the cheek went down, and the patient was again entirely free from all symptoms of the disease, except that *mysterious tic*—it still continued. And in that condition he remains to this day.

I have now related the treatment of three cases of tic douloureux, wherein the use of lunar caustic, so far as it is possible to determine, has effected cures. I will now give you a case wherein this same remedy failed to a certain extent.

About six weeks since, Mr. Mc——, of Pennsylvania, called on me with tic douloureux on the right side of his face; he had been affected with it for several years; thinks the disease was occasioned by a severe cold. The nerves involved were the first, second, and third branches of the fifth pair; *the third more than the first or second. In this particular, this case differed from all the preceding ones.* After perforating the antrum, and treating the case after the same manner I treated Mr. P——, the pain in the first and second branches subsided entirely; but there was little if any improvement in the condition of the third branch. This patient, however, was only under treatment five days before he left the city. I have since learned that he was not benefitted by the treatment in the slightest degree. This case does not, in my opinion, detract from the value of lunar caustic in the least, as a remedy for the cure of tic douloureux; it only shows that in cases where the third branch is involved, the caustic, to prove effective, must be applied to parts different from those I have usually selected.

I will now close my report of cases in which I have used lunar caustic, with one differing very materially from all the rest.

Mrs. C——, of Short Creek, in this county, was taken sick about the first of February last. Soon after the commencement of her sickness, she had a fearful spell of flooding, leaving her unable to arise from her bed for several weeks. During this sickness, she often experienced a great aching in the back part of her neck, close to the base of the skull. At last her head had to be arranged with great care upon the pillow, to avoid this kind of suffering. About two weeks after she first felt this pain, she was suddenly attacked with tic douloureux along the temple, and over the eye. The pain was of the most intense character. She was treated by her physicians with

*tonics*, *blisters*, *morphia*, and a host of other remedies, for two months and a half, with but little if any relief. She was finally brought to Wheeling. I found, by pressure over the first cervical vertebra, great soreness; and by pressure on one particular spot, pain in the temple was instantly produced. I now applied lunar caustic over the painful region of her neck very freely. Next day she complained very much of the soreness occasioned by the caustic, but the pain in the temple and neck was not so frequent, nor half so severe. I now applied caustic over the brow, and along the temple. The next day she was entirely free from all pain of a neuralgic character, and so she still remains.

I must not neglect to mention, that when she came to Wheeling, she was taking quinine and iron. These remedies I continued, except that I gave her the wine of iron in place of the sulphate of iron, which she was then taking.

In conclusion I have only to add, that of all the deductions that might be drawn from the history and treatment of the cases just reported, I leave entirely to the members of the society.

Wheeling, October 10, 1848.

